# Temporary Mental Nerve Paresthesia Originating from Periapical Infection

**DOI:** 10.1155/2015/457645

**Published:** 2015-08-05

**Authors:** Ozgur Genc Sen, Volkan Kaplan

**Affiliations:** ^1^Department of Endodontics, Faculty of Dentistry, Yüzüncü Yıl University, Kampus, 65080 Van, Turkey; ^2^Sakarya Oral and Dental Health Hospital, Ministry of Health, Sakarya, Turkey

## Abstract

Many systemic and local factors can cause paresthesia, and it is rarely caused by infections of dental origin. This report presents a case of mental nerve paresthesia caused by endodontic infection of a mandibular left second premolar. Resolution of the paresthesia began two weeks after conventional root canal treatment associated with antibiotic therapy and was completed in eight weeks. One year follow-up radiograph indicated complete healing of the radiolucent periapical lesion. The tooth was asymptomatic and functional.

## 1. Introduction

Paresthesia is defined as a burning or prickling sensation or partial numbness caused by neural injury [1]. In dentistry, paresthesia can be caused by systemic or local factors. Some of the systemic disorders that may cause orofacial paresthesia are multiple sclerosis [2], viral and bacterial infections [3, 4], and leukemia and lymphoma [5]. Local factors include anesthetic injections [6], surgical interventions [7], compressive phenomena or local infections [8, 9], and endodontic treatment [10, 11].

Paresthesia due to periapical infection may be caused by

1. mechanical pressure and ischemia related to the inflammatory process (edema) or its local pressure on the mental nerve resulting from accumulation of purulent exudate in the mandibular bone;
2. the toxic metabolic or inflammatory products of bacteria;
3. sufficient pressure from a subsequent hematoma [12–14].

Examination of the area affected by paresthesia can be carried out by thermal, mechanical, electrical, or chemical tests that elicit subjective responses. A more objective test is based on electrophysiologic analysis of the nerve. Radiographic and neurophysiologic screening is also required [14].

Treatments for paresthesia include removal of the cause and conservative (promotion of nerve regeneration) or surgical (nerve repair) procedures [15]. Antibiotics, nonsteroidal anti-inflammatory drugs, corticosteroids, proteolytic enzymes, and vitamin B are the drugs which can be used in addition to therapy [16].

We present a case of paresthesia involving the mental nerve as a result of periapical infection.

## 2. Case Presentation

A 20-year-old woman applied to Yüzüncü Yıl University Faculty of Dentistry with a complaint of severe spontaneous pain and swelling of the left mandible. The swelling and pain were associated with a total loss of sensitivity in the left side of the skin and mucosa of her lower lip.

The patient's general health was good, and she reported that she was not taking any medications.

In the extraoral examination, we noted lymphadenopathy and slight swelling on the skin corresponding to the inferior left second premolar area. The swelling was associated with warm red skin and pain.

Soft-tissue sensitivity was evaluated with a dental probe and an ice stick inserted into a plastic bag. The test revealed a complete loss of tactile, pain, and thermal sensation in the left inferior lip, including the skin on the right side of the chin and the oral mucosa up to the midline.

**Figure 1:**
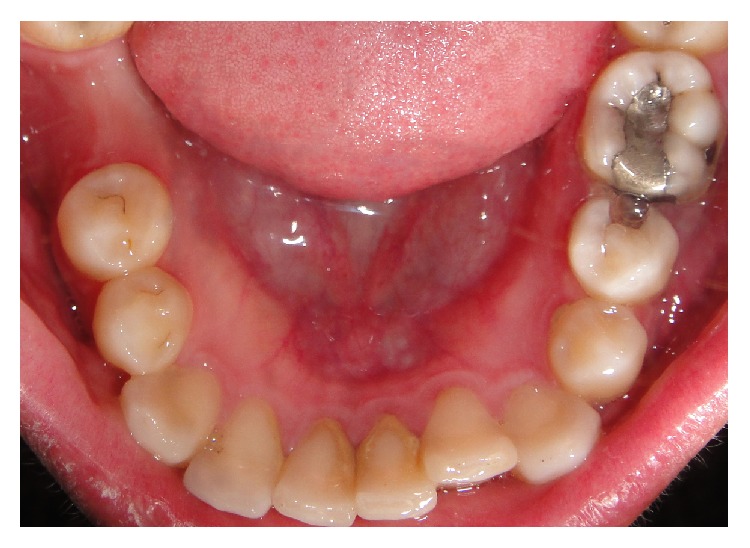
Preoperative intraoral view of the case.

**Figure 2:**
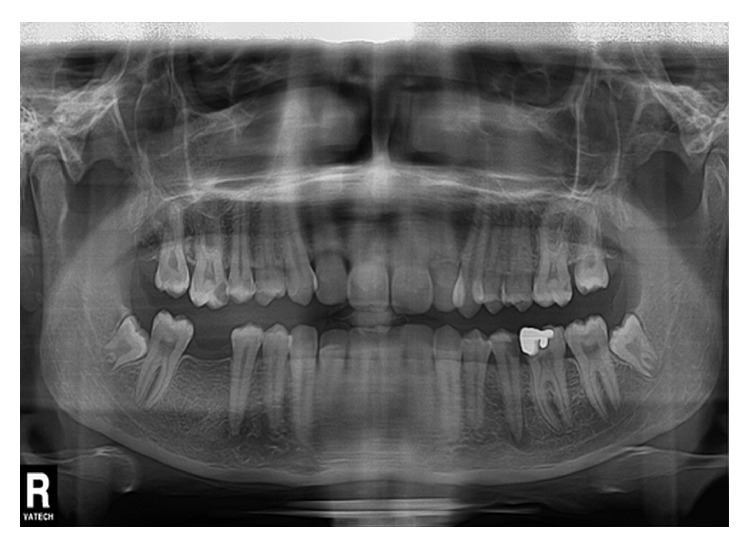
Preoperative panoramic radiograph of the case. Root of mandibular left second premolar seems to be in close proximity to mental nerve.

Intraoral examination revealed a swelling in the vestibular sulcus. The mucosa over the apical portion of the second premolar was extremely sensitive to palpation. Examination of the dentition revealed a caries lesion on the occlusal and distal surfaces of the left mandibular second premolar (Figure 1). The tooth was very sensitive to percussion. An electric pulp test did not provoke any response from the tooth. All adjacent teeth gave vital responses to the electric pulp test. Radiographic examination revealed the second mandibular premolar as having periapical radiolucency in close proximity to the mental foramen (Figure 2).

Even though the patient was exhibiting stress and supersensitivity, no intraoral treatment was performed that day. She was prescribed penicillin V (500 mg; 1 tablet four times a day for 5 days) and naproxen sodium (550 mg; 1 tablet twice a day for pain if needed). She was also given a prescription for vitamin B12 (1 tablet daily until paresthesia subsided).

The patient was seen after five days. Swelling and pain had completely disappeared. Local anesthesia was administered. Caries was removed and access to the endodontic cavity was opened by means of a diamond bur (Dentsply Maillefer, Tulsa, OK) with a high-speed handpiece. Only a small amount of exudate was present in the pulp chamber. The root canal was repeatedly irrigated with saline. Working length was determined using #15 K-files (Dentsply DeTrey, Konstanz, Germany) with an electronic apex locator (Romi Apex, Romidan, Israel) and confirmed by digital radiography. The canal was cleaned and shaped with Mtwo rotary files (VDW GmbH, Munich, Germany) to size 40, 0.06 taper, with intermittent saline irrigation. The root canal was dried with multiple paper points, and the tooth was provisionally sealed with a cotton pellet and Cavit G (3M ESPE, Seefeld, Germany) to allow for drainage.

**Figure 3:**
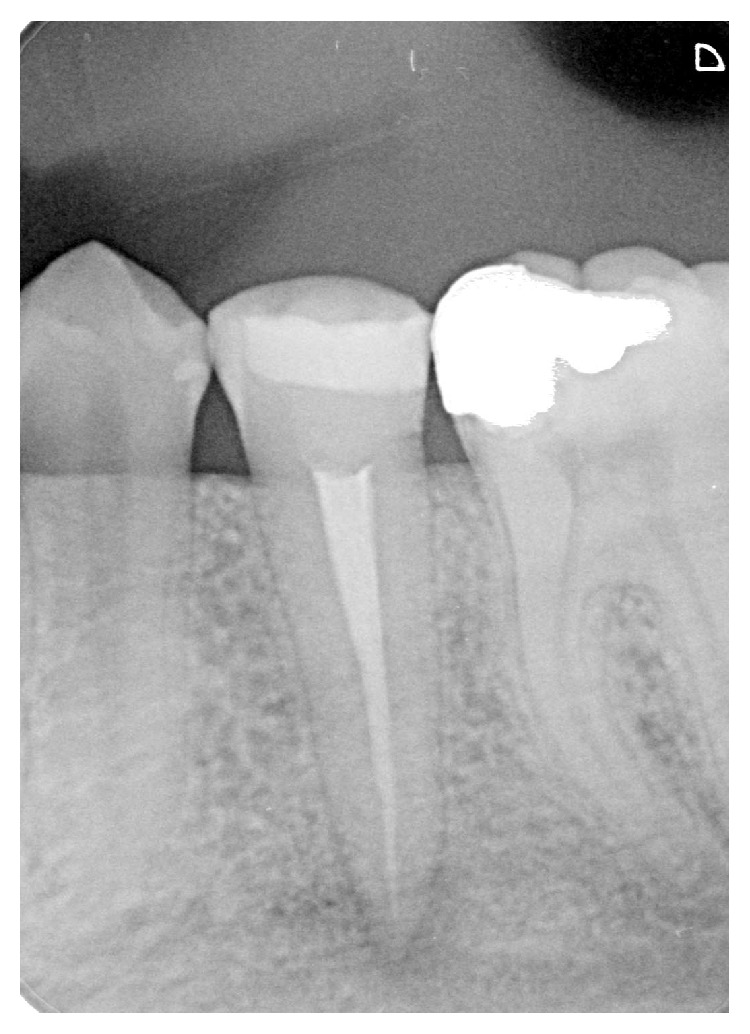
Periapical radiograph of mandibular left second premolar taken after root canal treatment.

**Figure 4:**
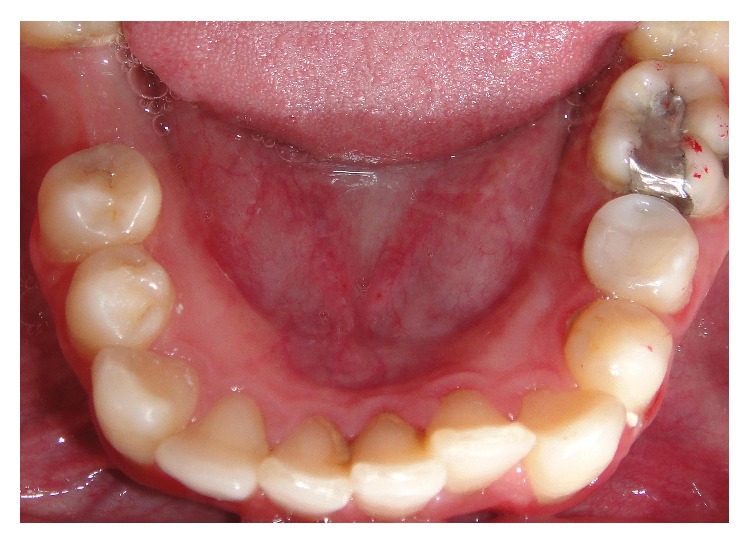
Clinical intraoral view of case after coronal restoration.

The patient was seen after two days. The pain was reduced, and swelling had partially subsided, but the paresthesia was still present. The temporary restoration was removed. No exudate was detected in the pulp chamber. A calcium hydroxide dressing was applied after multiple irrigations with NaOCl and drying procedures. The dressings were repeated in one week's time, to the complete resolution of paresthesia. The resolution began after two weeks and was completed in eight weeks. Subsequently, obturation was performed with gutta percha points (Mtwo, VDW GmbH) and AH plus sealer (Dentsply DeTrey, Zürich, Switzerland) (Figure 3). A hybrid composite (Filtek Z250, 3M ESPE) was used for coronal restoration (Figure 4).

**Figure 5:**
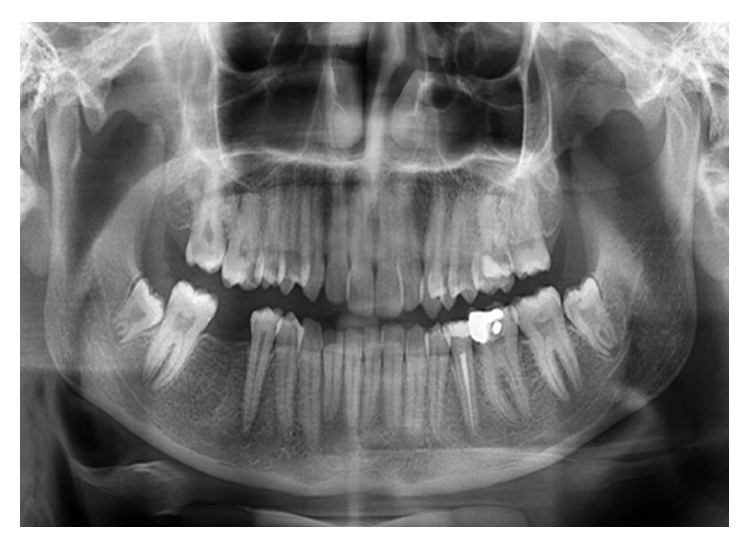
Follow-up panoramic radiograph taken after 1 year. Periapical lesion of mandibular second premolar is completely disappeared.

Since then, the patient did not keep appointments for control. One year later, we incidentally met her in another department of our faculty. A panoramic radiograph was taken. It indicated complete healing of the lesion (Figure 5). The patient was comfortable and asymptomatic, and the tooth was functional.

## 3. Discussion

The etiological factors involved in mental nerve paresthesia were analyzed, and it was concluded that the most common cause of mental nerve paresthesia (MNP) was invasive dental treatment (e.g., extractions, implants) [17]. It has also been reported that only 15% of the cases occurred due to an inflammatory process. A correspondingly limited number of cases of periapical-infection-related MNP have been reported [9, 12–14, 18–21].

At present, a diagnosis of sensory disturbances of mental nerve paresthesia is still mostly based on subjective clinical sensory testing, in common with inferior alveolar nerve paresthesia. It can be divided into two basic categories, mechanoreceptive and nociceptive, based upon the specific receptors stimulated through cutaneous contact. Mechanoreceptive tests include static light touch, two-point discrimination, and brush stroke direction. Pin tactile discrimination and thermal discrimination are nociceptive tests [15]. In the case reported here, we evaluated tissue sensitivity by touching the affected area with the tip of a dental probe and an ice stick inserted into a plastic bag.

Infection-related MNP usually subsides after appropriate endodontic therapy or surgical interventions. As soon as the cause is removed, paresthesia should resolve within days or weeks. Morse [12] reported a case of mental paresthesia due to infection of the mandibular first premolar in which paresthesia began one day after initial endodontic treatment. Paresthesia was treated by irrigation, antibiotics, and dexamethasone and was completely resolved in seven weeks. Naik et al. [20] reported the resolution of MNP due to endodontic infection of the mandibular second premolar within one week after endodontic treatment. In our case, reported here, conventional endodontic treatment, supported with prescription antibiotics, resolved paresthesia in eight weeks.

In paresthesia cases, because of the close proximity of the lesion and the nerve, a more conservative treatment should be the first choice. However, if it is deemed necessary, surgical intervention should not be delayed, because the longer the mechanical or chemical irritation persists, the more the nerve fibers degenerate and the greater the risk of paresthesia is becoming permanent [22]. Also, uncontrolled infection can lead to serious complications. Cohen et al. [23] reported a case of paresthesia that led to meningitis. In the present case, we initiated the treatment procedures immediately to prevent further damage, and resolution of paresthesia began in two weeks.

## 4. Conclusion

Periapical infections of mandibular premolars can lead to serious neurological complications due to their proximity to mental foramen. A conventional endodontic therapy can be a simple and conservative choice for the treatment.
